# Comparative transcriptome analysis of *Armillaria gallica* 012m in response to ethephon treatment

**DOI:** 10.7717/peerj.14714

**Published:** 2023-01-17

**Authors:** Haiying Yang, Kaixiang He, Yapu Cao, Zhihao Li, Qiaolin Ji, Jingxian Sun, Ganpeng Li, Xin Chen, Haiying Mo, Gang Du, Qingqing Li

**Affiliations:** 1 Yunnan Minzu University, School of Chemistry and Environment, Kunming, Yunnan, China; 2 Yunnan Minzu University, Key Laboratory of Chemistry in Ethnic Medicinal Resources Ministry of Education, Kunming, Yunnan, China; 3 Southwest Forestry University, Life Science College, Kunming, Yunnan, China; 4 Kunming Xianghao Technology Co. Ltd, Kunming, Yunnan, China

**Keywords:** RNA-Seq, ET, ETH, Response, Differential expression genes, ET receptor

## Abstract

**Background:**

* Gastrodia elata,* known as a rootless, leafless, achlorophyllous and fully mycoheterotrophic orchid, needs to establish symbionts with particular* Armillaria* species to acquire nutrition and energy. Previous research findings had approved that ethylene (ET) played an important role in plant-fungi interaction and some receptors of ET had been discovered in microorganisms. However, the molecular mechanisms underlying the role of ET in the interaction between *G. elata* and* Armillaria* species remain unknown.

**Methods:**

Exiguous ethephon (ETH) was added to agar and liquid media to observe the morphological features of mycelium and count the biomass respectively. Mycelium cultured in liquid media with exiguous ETH (0.1 ppm, 2.0 ppm, 5.0 ppm) were chosen to perform whole-transcriptome profiling through the RNA-seq technology (Illumina NGS sequencing). The DEGs of growth-related genes and candidate ET receptor domains were predicted on SMART.

**Results:**

ETH-0.1 ppm and ETH-2 ppm could significantly improve the mycelium growth of *A. gallica* 012m, while ETH-5 ppm inhibited the mycelium growth in both solid and liquid media. The number of up-regulated or down-regulated genes increased along with the concentrations of ETH. The growth of mycelia might benefit from the up-regulated expression of Pyr_redox (Pyridine nucleotide-disulphide oxidoreductase), GAL4 (C6 zinc finger) and HMG (High Mobility Group) genes in the ETH-0.1 ppm and ETH-2 ppm. Therefore, the growth of mycelia might be impaired by the down-regulated expression of ZnF_C2H2 and ribosomal protein S4 proteins in the ETH-5 ppm. Seven ET receptor domains were predicted in *A. gallica* 012m. Based on cluster analysis and comparative studies of proteins, the putative ETH receptor domains of* A. gallica* 012m have a higher homologous correlation with fungi.

**Conclusions:**

The responses of *A. gallica* 012m to ETH had a concentration effect similar to the plants’ responses to ET. Therefore, the number of up-regulated or down-regulated genes are increased along with the concentrations of ETH. Seven ET receptor protein domains were predicted in the genome and transcriptome of *A. gallica* 012m. We speculate that ETH receptors exist in *A. gallica* 012m and ethylene might play an important role in the plant-fungi interaction.

## Introduction

*Armillaria* species (Basidiomycota, Physalacriaceae) are well-known as plant pathogens that cause serious root diseases on woody plants in forests and plantations (Sipos et al., 2017; Gasic et al., 2021). However, several species of *Armillaria* have been confirmed to engage in symbiotic associations with various plants, insects and other fungi (Koch & Herr, 2021; Liang et al., 2021). *Gastrodia elata*, known as a rootless, leafless, achlorophyllous and fully mycoheterotrophic orchid (Kikuchi & Yamaji, 2010), needs to establish symbionts with particular *Armillaria* species to acquire nutrition and energy (Cha & Igarashi, 1995; Guo et al., 2016; Yuan et al., 2018). The plant-fungi interaction has long attracted the interest of botanists and microbiologists. Previous research findings had approved that phytohormones played an important role in plant-fungi interaction (Chanclud & Morel, 2016; Eichmann, Richards & Schäfer, 2021).

ET is often recognized as the senescence hormone involved in many aspects of plant physiology and development (Carlew, Allen & Binder, 2020). In the same time, ET seems to be an early plant defense factor in infected plants and influences both the plant and the plant pathogen (Chague et al., 2006). In addition to plants, several bacteria and fungi can produce and perceive ET (Arshad & Frankenberger, 1991; North et al., 2017). The response of fungi to ET is multifarious depending on the characteristics of the species and ET concentration (Chague et al., 2006). Previous studies showed exogenous ET improved spore germination and mycelium growth of *Alternaria alternate*, *Penicillium digitatum*, *P. italicum* and *Thielaviopsis paradox*, while inhibiting spore germination and hyphae elongation of Botrytis cinerea (El-Kazzaz, 1983; Flaishman & Kolattukudy, 1994; Kępczyńska, 1994; Carlew, Allen & Binder, 2020). In other cases, exogenous ET influence the colonization of mycorrhizal fungi and the formation of nodules (Foo et al., 2016; Bedini et al., 2018; Carlew, Allen & Binder, 2020).

ETH was used as an ethylene-generating agent (Lee, Holdo & Muzika, 2021). During our cultivation of *A. gallica* 012m, a low concentration of exogenous ETH improved the growth of mycelia, while a high concentration of exogenous ETH inhibited the growth of mycelia in both solid and liquid media. In our previous work, the draft genome of *Armillaria* strain 012m had been investigated (Zhan et al., 2020). This work provides the genome-wide transcriptional profiling to investigate the responses of *A. gallica* 012m to ETH, and discusses the role of ET in the plant-fungi interactions.

## Materials and Methods

### Fungi growth and culture conditions

*A. gallica* 012m was derived from *G. elata* in our previous research. The stock strain was routinely grown on modified Czapek-Dox medium agar (MgSO_4_, 0.5 g; FeSO_4_, 0.01 g; KCl, 0.5 g; NaNO_3_, 3 g; K_2_HPO_4_, 1 g; sucrose, 30 g; malt extract, 10 g; yeast extract, 10 g; ethanol, 20 g) in the dark at 25 °C for 15 days (Zhan et al., 2020). Exiguous ETH (0.1 ppm, 2.0 ppm, 5.0 ppm) were added to the medium of broth and ager; and set up the BK (blank control) groups. Setting three parallel repeats for each different treatment condition.

The mycelium of *A. gallica* 012m was grown on liquid media in a 160 r/min shaker at 25 °C for 15 days. Then, the pellets were filtrated with a Buchner funnel, washed with pure water, collected in beaker and weighted with electronic balance. By utilizing independent sample *T*-test, the data analysis was performed with IBM SPSS v23 (Tanner-Smith & Tipton, 2014). The mycelium was preserved under liquid nitrogen conditions.

### RNA extraction and sequencing

The method was as same as our previous work (Cao et al., 2022). RNA was extracted from fungi pellets using the RNeasy mini kit (Qiagen, Hiden, Germany) following the manufacturer’s instructions. The cDNA library was sequenced on the Illumina HiSeq platform (Caporaso et al., 2012) with a double-end sequencing strategy in Novogene Bioinformatics Technology, Beijing, China. The original data were deposited in the National Center for Biotechnology Information database with the accession number PRJNA759758.

### Transcriptome sequences data quality control and comparison

To obtain clean transcriptome data, low-quality sequences of RNA-seq were removed with Trimmomatic v0.36 (Bolger, Lohse & Usadel, 2014). Then, the data quality control was evaluated with FastQC v0.11.9 (Brown, Pirrung & McCue, 2017). The reads of RNA-Seq was aligned with the *A. gallica* 012m genome sequences (https://www.ncbi.nlm.nih.gov/genome/57439?genome_assembly_id=853036) by using Hisat2 v2.1.0 (Kim et al., 2019) to obtain the SAM data.

### Differentially expressed gene analysis

Gene expression values were performed according to the reads per kilobase per million mapped reads (RPKM) method. The read count matrix was obtained for expression qualification with StringTie v2.1.0 (Pertea et al., 2015). The read count matrix was imported into R 3.6.3. The read count matrix was imported into R 3.6.3, and the differential gene analysis was carried out with edgeR of R package under an FDR<0.05 and —log2FC—>2. Next, RNASeqPower (DOI: http://dx.doi.org/10.18129/B9.bioc.RNASeqPower), a power analysis calculation software, was used to calculate the statistical power of this experimental design, and the statistical power is 0.81. Then, all transcripts and their corresponding genes were compared by emapper v2.1.3 for functional annotation and classification (Huerta-Cepas et al., 2017). The result of the GO function analysis was performed by using TBtools V0.66836 (Chen et al., 2020). Go terms visualization of DEGs was executed with WEGO (https://wego.genomics.cn/).

### Functional analysis of growth-related genes of DEGs

To analyze the function of protein domain, the gene sequences involved biological process functions were extracted and their protein domains were annotated by using the SMART platform (http://smart.embl-heidelberg.de/).

### Screened candidate ethylene receptors

Those existing ET receptor protein sequence of fungi were downloaded the from NCBI (https://www.ncbi.nlm.nih.gov/) and compared with the genome protein sequences of *A. gallica* 012m. Those expressed sequences with *E*-value ≤ le-5 were selected and detected their conserved domain on SMART (Letunic, Khedkar & Bork, 2021).

### Construction of receptor sequence evolutionary tree

An evolutionary tree was constructed with downloaded receptor sequences and screened sequences of *A. gallica* 012m. ClustalW2 of MEGA7 was used for multiple amino acid sequences alignment (Kumar, Stecher & Tamura, 2016). The phylogenetic tree was constructed by neighbor-joining method and the number of calculations was 1,000.

## Results

### Morphological characteristics of *A. gallica* grown under ETH

**Figure 1:**
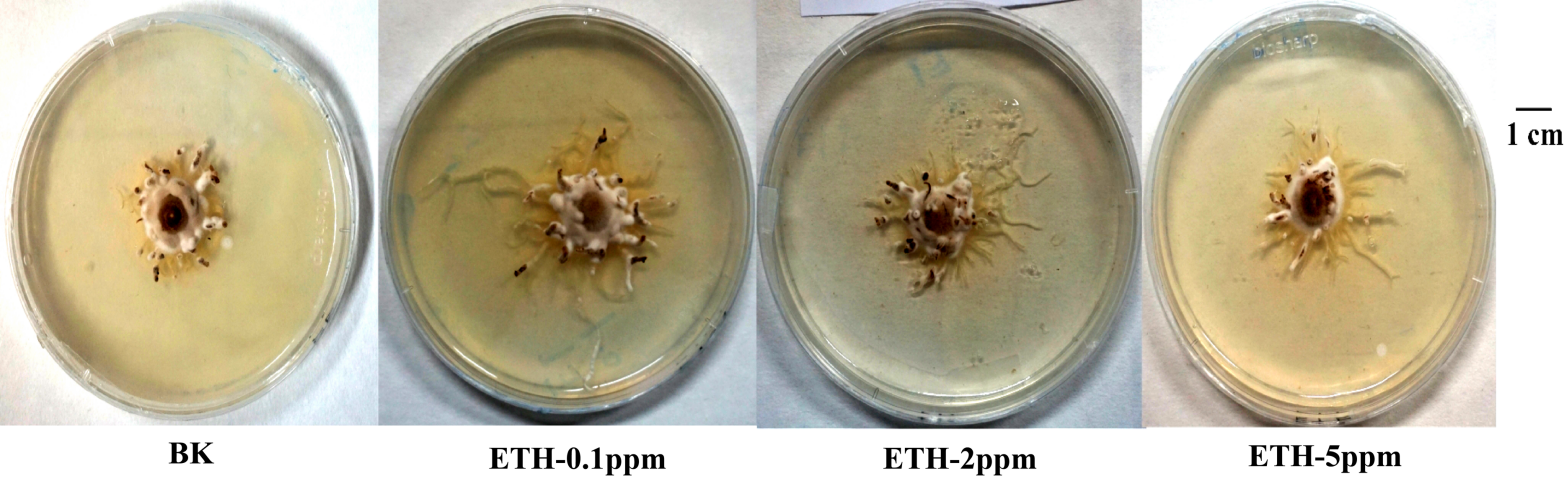
Growth status of *A. gallica* 012 m in ETH (0.1 ppm, 2.0 ppm, 5.0 ppm) and BK.

As shown in Fig. 1, ETH stimulated the mycelial growth of *A. gallica* 012m in solid media. Moreover, lower concentrations of ETH present better effect on mycelium elongation of *A. gallica* 012m. The order of influence of mycelium elongation were ETH−0.1 ppm>ETH-2 ppm>ETH-5 ppm.

### Effects of plant growth substances on the biomass of *A. gallica* 012m

**Figure 2:**
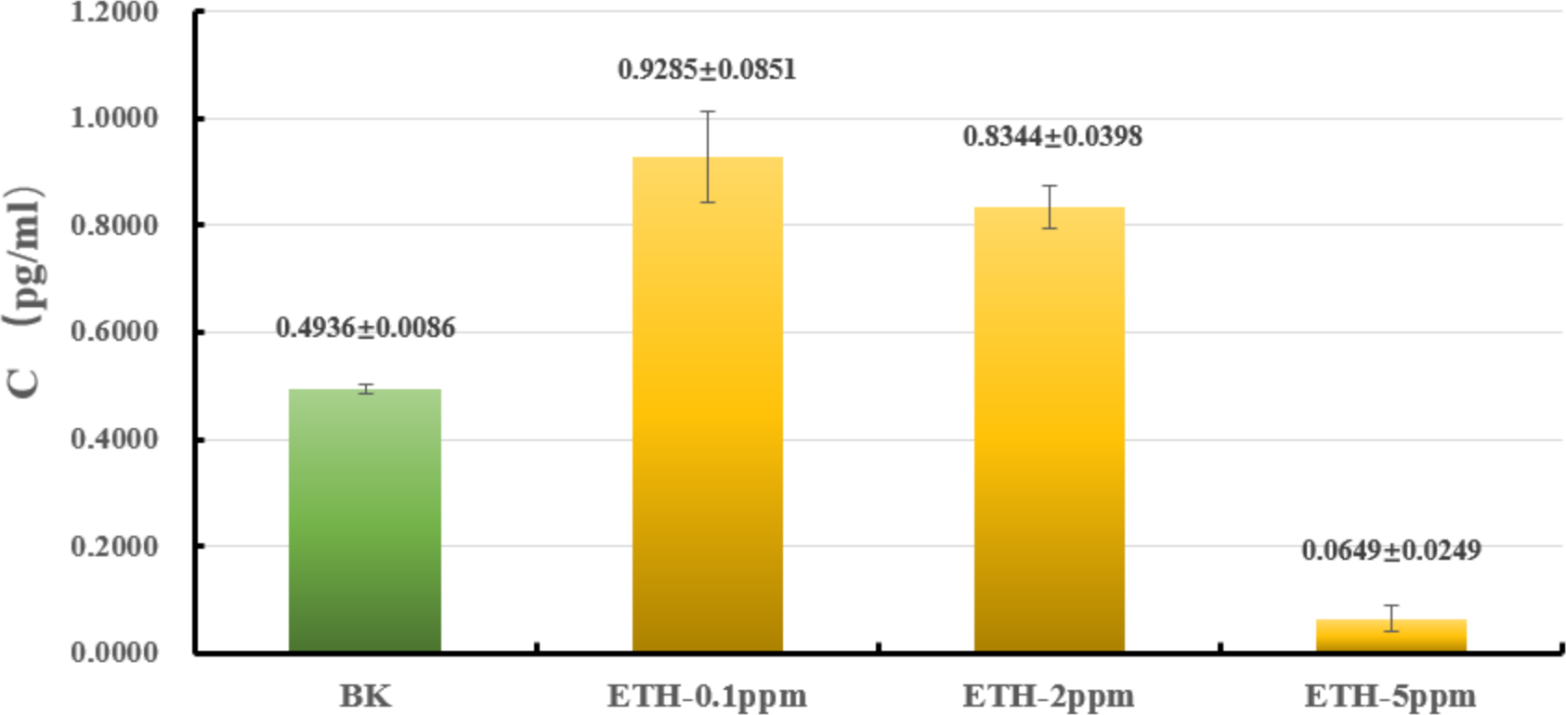
Effects of ETH on biomass of *A. gallica* 012m. The error line represents the standard deviation.

The liquid culture was carried out to explore whether or not ETH affected the biomass of *A. gallica* 012m. As shown in Fig. 2 and Table S1, the biomass of mycelium increased by 88.0 ± 9.1% under ETH−0.1ppm, the biomass of mycelium increased by 66.1 ± 7.9% under ETH-2ppm, the biomass of mycelium decreased by 86.8 ± 5.0% under ETH-5ppm. By utilizing independent sample *T*-test, it can be considered that the biomass of *A. gallica* 012m were increased extremely significant (*p* < 0.01) under ETH−0.1 ppm and ETH-2ppm, while decreased extremely significant (*p* < 0.01) under ETH-5ppm.

### Evaluation of transcriptome sequencing data

Above 10.0 Gb of raw data per sample was obtained by transcriptome sequencing on the Illumina HiSeq platform and could be used to further expression level analysis after quality control. In addition, twelve transcriptome samples of ETH and BK were sequenced by Illumina HiSeq platform to obtain 21,016,459; 25,437,814; 22,314,980; 26,576,772; 24,979,536; 23,865,405; 30,433,600; 21,398,103; 22,659,153; 22,659,153; 27,395,065; 28,784,179 and 26,125,609 pairs of PE reads (Table 1).

**Table 1:** Summary of sequencing data for 12 samples of ETH and BK.

Sample_name	*Clean_reads*	*Read mapped (%)*	*Total mapped*	*Multiple mapped*	*Uniquelymapped*
ETH-0.1 ppm-1	21,016,459	92.73	20,029,878	763,543	19,266,335
ETH-0.1 ppm-2	25,437,814	92.85	24,371,998	735,341	23,636,657
ETH-0.1 ppm-3	22,314,980	92.89	21,296,185	611,455	20,684,730
ETH-2 ppm-1	26,576,772	93.64	25,535,583	686,444	24,849,139
ETH-2 ppm-2	24,979,536	93.82	23,963,179	670,682	23,292,497
ETH-2 ppm-3	23,865,405	92.58	22,673,985	729,855	21,944,130
ETH-5 ppm-1	30,433,600	93.76	29,253,416	872,677	28,380,739
ETH-5 ppm-2	21,398,103	93.64	20,536,212	670,720	19,865,492
ETH-5 ppm-3	22,659,153	93.69	21,758,215	703,163	21,055,052
BK-1	27,395,065	93.06	25,970,049	871,066	25,098,983
BK-2	28,784,179	93.24	27,459,809	780,605	26,679,204
BK-3	24,916,601	93.27	23,812,273	645,411	23,166,862

After removing the reads with adapter sequences of low quality, an average of 21,916,601 to 30,433,600 pairs of clean reads were retained from ETH and BK, respectively. Read mapped percentage of clean data from all sample were higher than 92.73%, and reads mapped percentages of ETH-2ppm-2 was the highest. What’s more, 92.73% ∼93.82% pure readings were successfully mapped to the reference genome of *A. gallica* 012m.

### Enriched GO terms of up-regulatedand down-regulated at DEGs

**Figure 3:**
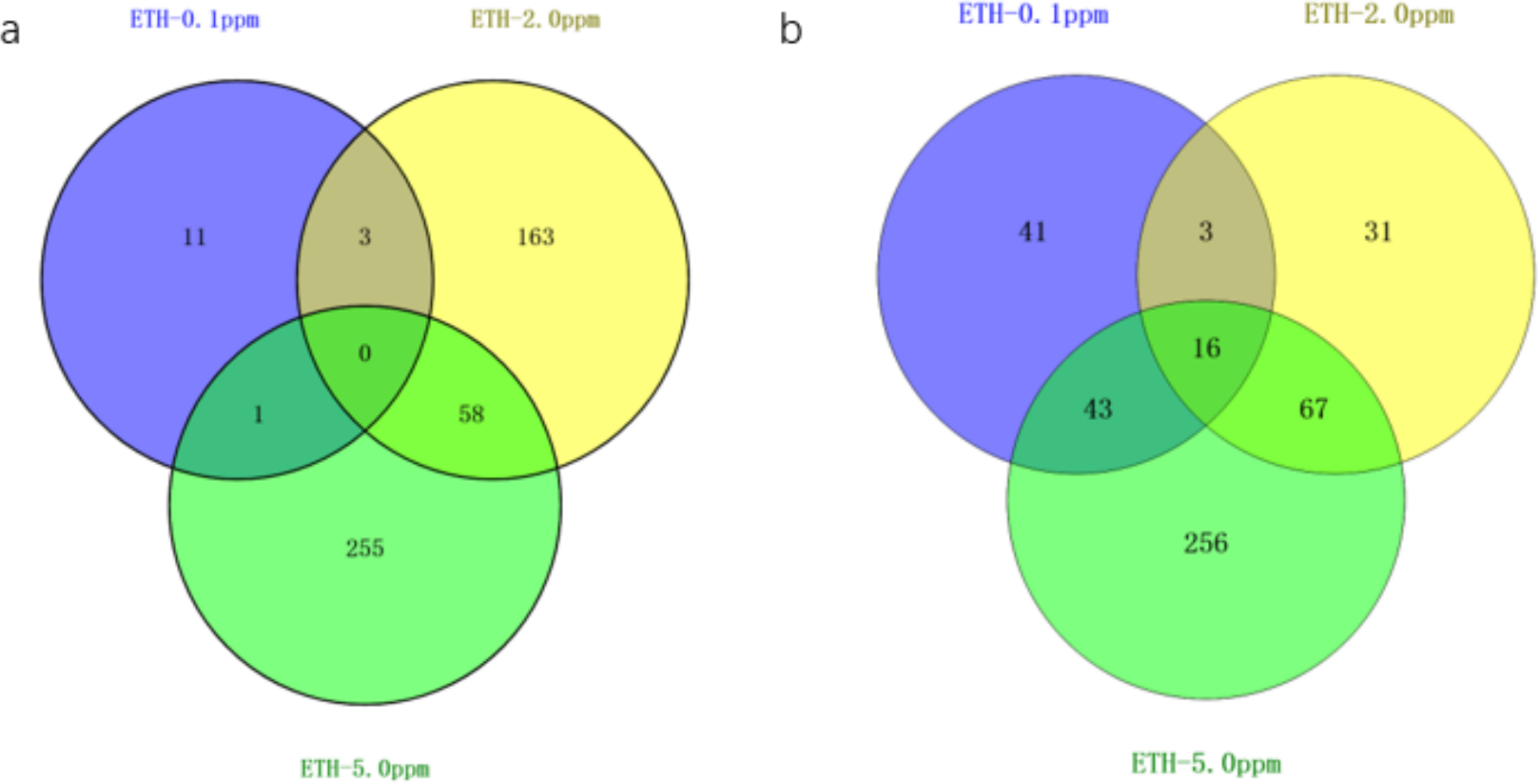
Venn diagram of differentially expressed genes ETH (0.1 ppm, 2.0 ppm and 5.0 ppm). (A) Venn diagram showing overlapping DEGs up-regulated in response to ETH (0.1 ppm, 2.0 ppm and 5.0 ppm). (B) Venn diagram showing overlapping DEGs down-regulated in response to ETH (0.1 ppm, 2.0 ppm and 5.0 ppm).

In the ETH−0.1 ppm *vs.* BK comparison, a total of 118 genes were differentially expressed, including 15 up-regulated genes and 103 down-regulated genes (Fig. 3, Table S2). In the ETH-2 ppm *vs.* BK comparison, a total of 341 genes were differentially expressed, including 224 up-regulation genes and 117 down-regulation genes (Table S3). In the ETH-5 ppm *vs.* BK comparison, a total of 696 genes were differentially expressed, including 314 up-regulated genes and 382 down-regulated genes (Table S4). Interestingly, the total number of DEGs in the experimental group increased along with ETH concentration.

**Figure 4:**
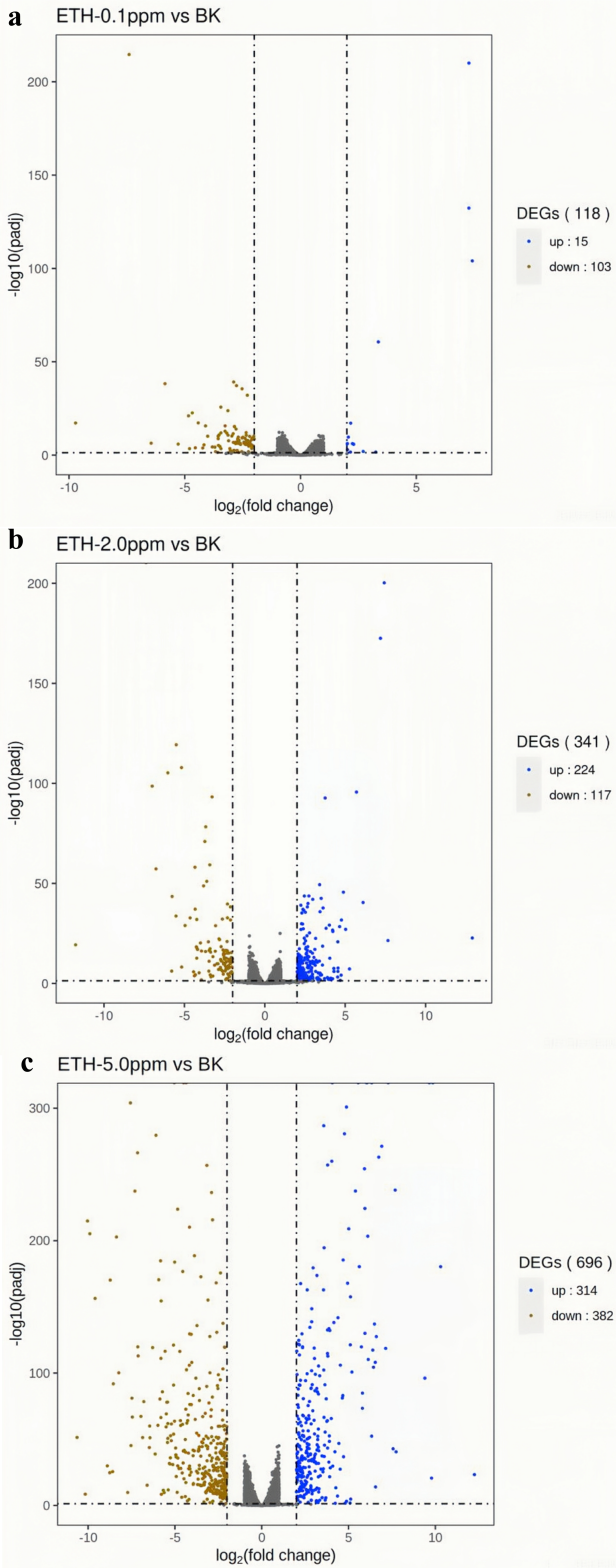
(A–C) Volcano map of differentially expressed genes. Differentially expressed genes (DEGs) were defined by edgeR with an FDR < 0.05 and —log2FC—>2 and corrected *p* value (padj) < 0.05.

The gene expression of *A. gallica* 012m with ETH−0.1 ppm *vs.* BK, ETH−2.0 ppm and ETH−5.0 ppm *vs.* BK was analyzed. In the up-regulated genes, the results showed that there were three differential genes shared by ETH−0.1 ppm and ETH−2.0 ppm, and 58 differential genes shared by ETH−2.0 ppm and ETH−5.0 ppm, 11 DEGs in ETH−0.1 ppm group alone, 163 DEGs in ETH−2.0 ppm group alone, and 255 DEGs in ETH−5.0 ppm group alone. In down-regulation genes, the DEGs of the ETH−0.1 ppm group were the same as the ETH−2.0 ppm group more than 15% (Fig. 4).

### Enriched GO terms of up-regulated and down-regulated at DEGs

**Figure 5:**
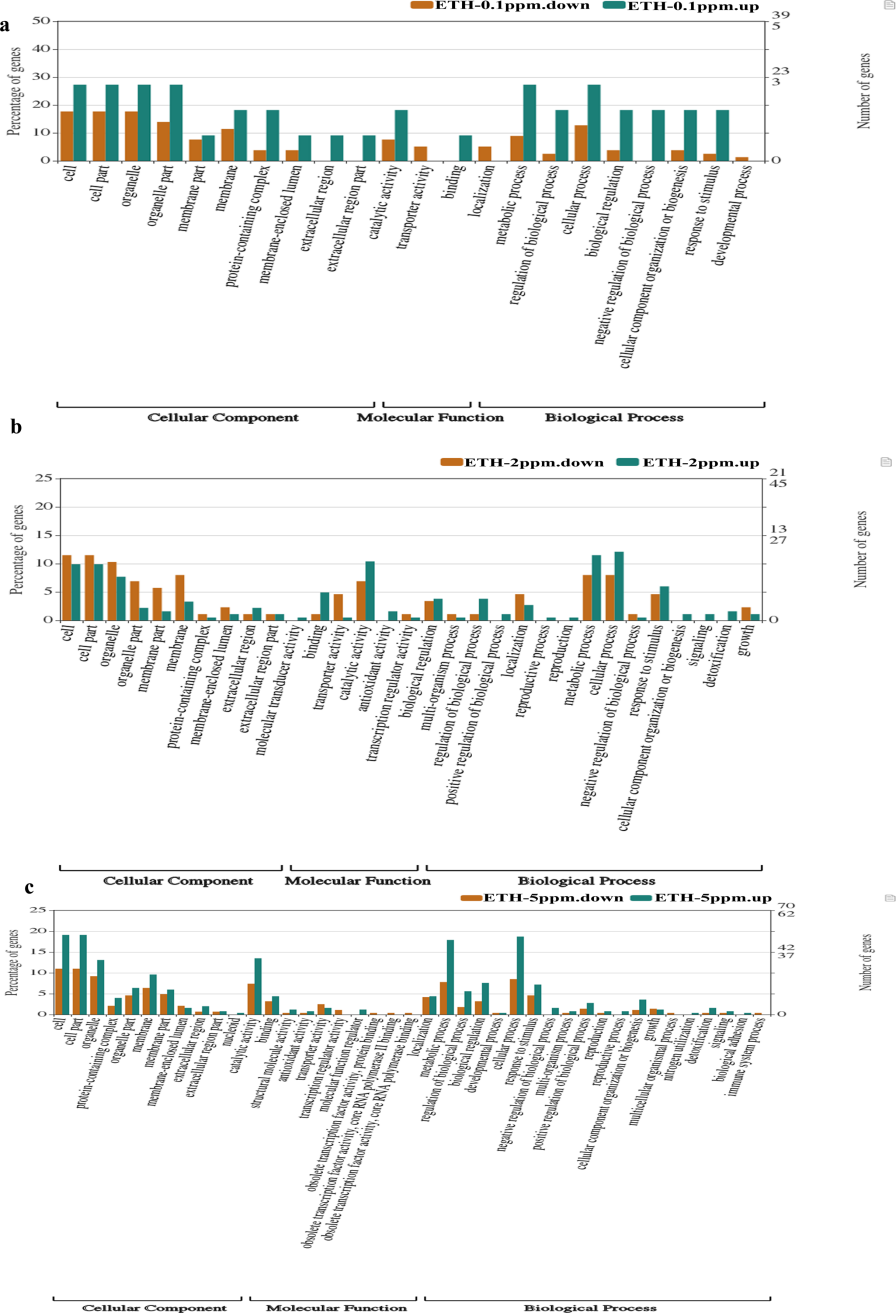
(A–C) Gene Ontology (GO) enrichment analysis of differentially expressed genes (DEGs).

Comparing with the GO database, classification and functional analysis of DEGs were performed for better visualization. The result of the annotation of Level 2 for the GO database was shown in Fig. 5.

At ETH−0.1 ppm *vs* BK, a total of 90 DEGs were categorized into 22 functional groups under CC (10 functional groups), MF (three functional groups) and BP (nine functional groups). In the CC category, the major terms included ‘cell’ (upregulation: downregulation = 3: 14), ‘cell part’ (upregulation: downregulation = 3: 14), ‘organelle’ (upregulation: downregulation = 3: 14), ‘organelle part’ (upregulation: downregulation = 3:11) and ‘membrane’ (upregulation: downregulation = 2: 9). In the MF category, the GO terms only included three functional groups, which were ‘catalytic activity (upregulation: downregulation = 2: 6), ‘transporter activity (upregulation: downregulation = 0: 4) and ‘binding’ (upregulation: downregulation = 1: 0). In the BP category, the representative GO terms included ‘cellular process’ (upregulation: downregulation = 3: 10), ‘metabolic process’ (upregulation: downregulation = 3: 7), ‘biological process’, ‘response to stimulus’ (upregulation: downregulation = 3: 2) and ‘regulation of biological process’ (upregulation: downregulation = 3: 2).

At ETH-2 ppm *vs* BK, a total of 269 DEGs were categorized into 34 functional groups under CC (10 functional t groups), MF (nine functional groups) and BP (15 functional groups). In the CC category, the dominant GO terms included ‘cell’ (upregulation: downregulation = 18: 10), ‘cell part’ (upregulation: downregulation = 18: 10), ‘organelle’ (upregulation: downregulation = 14: 9), ‘organelle part’ (upregulation: downregulation = 4: 7) and ‘membrane’ (upregulation: downregulation = 6: 7). In the MF category, the major GO terms included ‘catalytic activity (upregulation: downregulation = 19: 6), ‘transporter activity’ (upregulation: downregulation = 4: 1), ‘binding’ (upregulation: downregulation = 9: 1) and ‘antioxidant activity’ (upregulation: downregulation = 3: 0). In the BP category, the major GO terms included ‘metabolic process’ (upregulation: downregulation = 21: 7), ‘cellular process’ (upregulation: downregulation = 22: 7), ‘response to stimulus’ (upregulation: downregulation = 11: 4), ‘localization’ (upregulation: downregulation = 5: 4) and ‘biological regulation’ (upregulation: downregulation = 7: 3).

At ETH-5 ppm *vs.* BK, a total of 534 DEGs were categorized into 41 functional groups under CC (11 functional groups), MF (10 functional groups) and BP (20 functional groups). In the CC category, the major GO terms included ‘cell’ (upregulation: downregulation = 48: 31), ‘cell part’ (upregulation: downregulation = 48: 31), ‘organelle’ (upregulation: downregulation = 33: 26), ‘organelle part’ (upregulation: downregulation = 16: 13) and ‘membrane’ (upregulation: downregulation = 24: 18). In the MF category, the representative GO terms included ‘catalytic activity (upregulation: downregulation = 34: 21), ‘transporter activity’ (upregulation: downregulation = 4: 7), ‘binding’ (upregulation: downregulation = 11: 9) and ‘antioxidant activity’ (upregulation: downregulation = 2: 1). In the BP category, the main GO terms included ‘metabolic process’ (upregulation: downregulation = 45: 22), ‘cellular process’ (upregulation: downregulation = 47: 22), ‘biological regulation’ (upregulation: downregulation = 19: 9) and ‘regulation of biological process’ (upregulation: downregulation = 14: 5). These result demonstrated that the main biological functions of the genes expressed in the *A. gallica* 012m transcriptome.

### Identification of genes involved in the growth of *A. gallica* 012m

To further explore the mechanism of effects on the growth of *A. gallica* 012m, we respectively compared the biological regulation process of DEGs. The result can be shown as follows: in the ETH−0.1 ppm *vs* BK, Pyr_redox (pyridine nucleotide-disulphide oxidoreductase) domain was predicted in Armga012mGene24786 which showed up-regulation expressed. This domain is actually a small NADH binding domain within a larger FAD binding domain. This domain exists in NADH oxidases, peroxidases, class I and class II oxidoreductases. In the ETH-2 ppm *vs.* BK, HMG (High Mobility Group) and GAL4 (C6 zinc finger)/Fungal_trans domain were predicted in Armga012mGene25682 and Armga012mGene16364, respectively and showed up-regulation expressed. HMG-box domains form a large, diverse family involved in the regulation of transcription, replication and strand repair. Gal4 is a positive regulator for the gene expression of the galactose-induced genes of *S. cerevisiae*. This domain is found in various fungal transcription factors, which regulate cellular and metabolic processes. In the ETH-5 ppm *vs.* BK, ZnF_C2H2 (C2H2 zinc finger) and ribosomal protein S4 domain were predicted in Armga012mGene07208 and Armga012mGene07627 which showed down-regulation expressed. Znf-containing proteins function in gene transcription, translation, mRNA trafficking, cytoskeleton organisation, epithelial development, cell adhesion, protein folding, chromatin remodelling and zinc sensing. Ribosomal protein S4 is a kinds of small proteins that may be involved in translation regulation.

### Prediction and expression of ET receptors in *A. gallica* 012m

**Figure 6:**
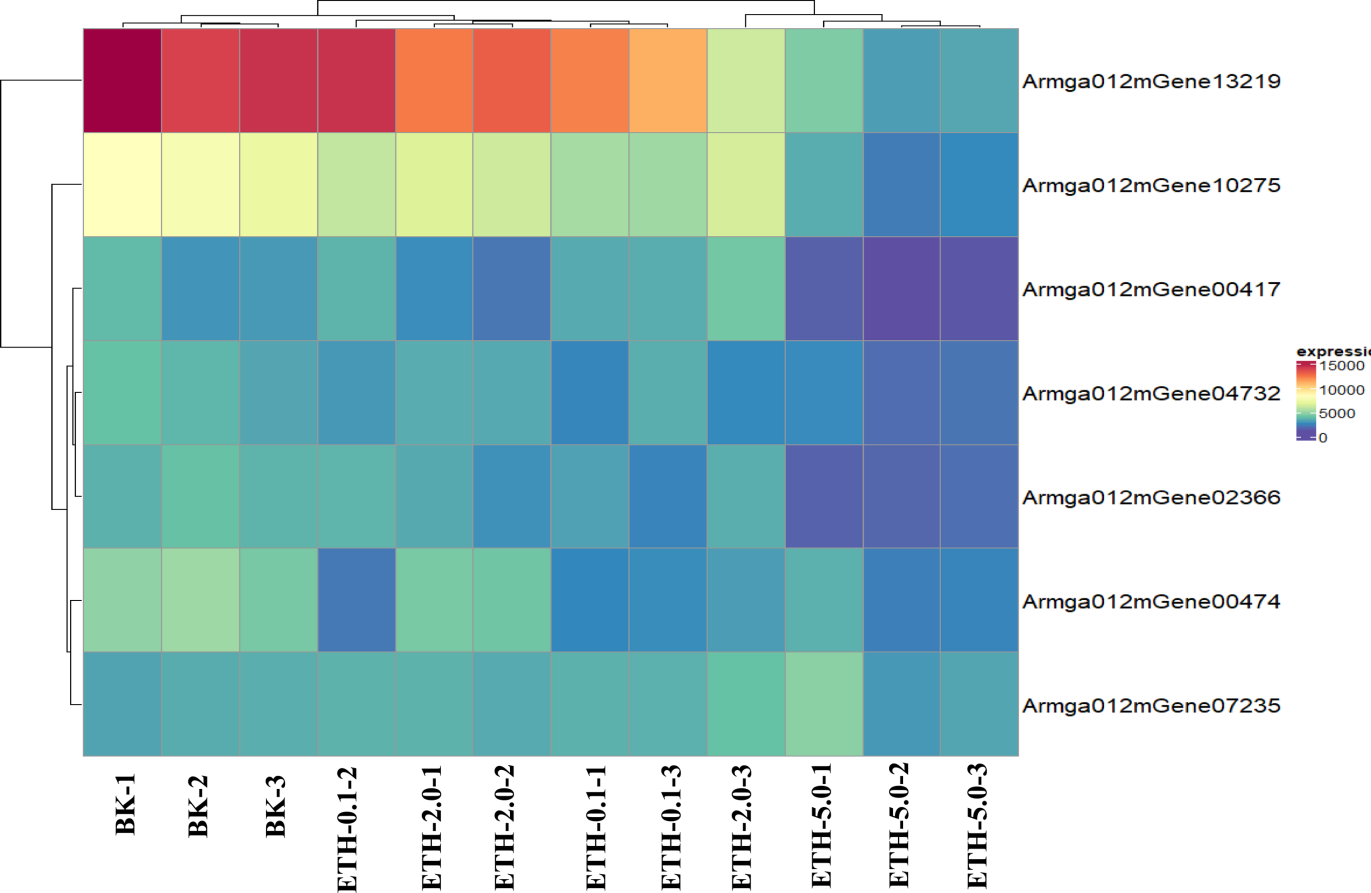
Expression pattern of selected clusters of genes containing ET receptor domain. The color intensity of each gene is based expression of genes containing ET receptor domain in BK samples and ETH (0.1 ppm, 2.0 ppm and 5.0 ppm) samples.

ET receptor is the first element of the ethylene biological effect, and its binding with ethylene can activate downstream ethylene signal transduction. We downloaded the sequence information of ET receptors of fungi from GenBank with a total of 54 sequences, compared it with the genome protein sequence of *A. gallica* 012m. A total of seven speculated ET receptor domains of *A. gallica* 012m were predicted by using SMART (Table 2) and expressed. The predicted ET receptor proteins were annotated, and only Armga012mGene13219 has transmembrane region domains. Those predicted ET proteins have similar domain as determined ethylene receptor proteins, and the expression levels of candidate ethylene receptor protein sequences were different (Fig. 6) in *A. gallica* 012m. Thus, we speculated that exogenous ethylene affected the growth of *A. gallica* 012 m through ET receptors.

**Table 2:** ET receptor protein and domain of *A. gallica* 012m.

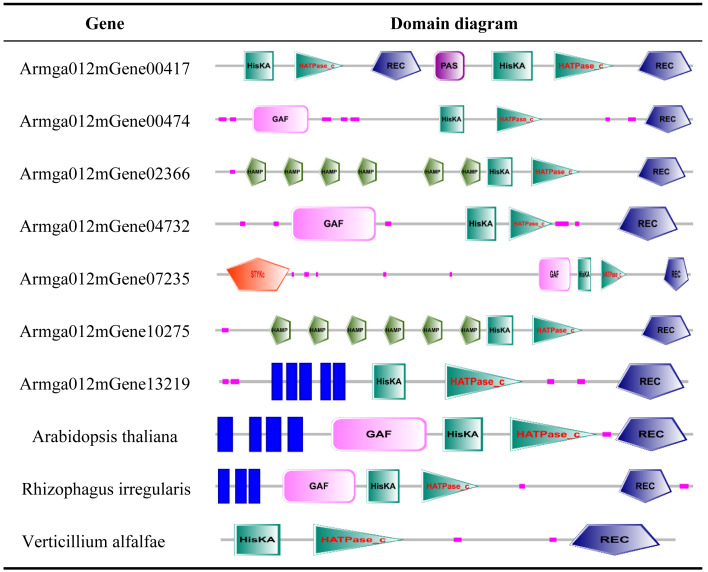

**Notes.**

ER endoplasmic reticulum HisKA Histidine Kinase A (dimerisation and phosphoacceptor) domain HATPase_c Histidine kinase-like ATPase catalytic (Histidine kinase-, DNA gyrase B-, phytochrome-like ATPases) domain GAF cGMP-phosphodiesterase, adenylyl cyclase, FhIA domain P PAS/PAC (Per-period circadian protein; Arnt-Ah receptor nuclear translocator protein Sim-single minded protein/PAS C terminus) REC Receiver domain GGDEF diguanylate cyclase domain EAL c-di-GMP phosphodiesterase STYKc protein kinase–unclassified specificity.

### Genes containing ET receptor domain in *A. gallica* 012m

**Figure 7:**
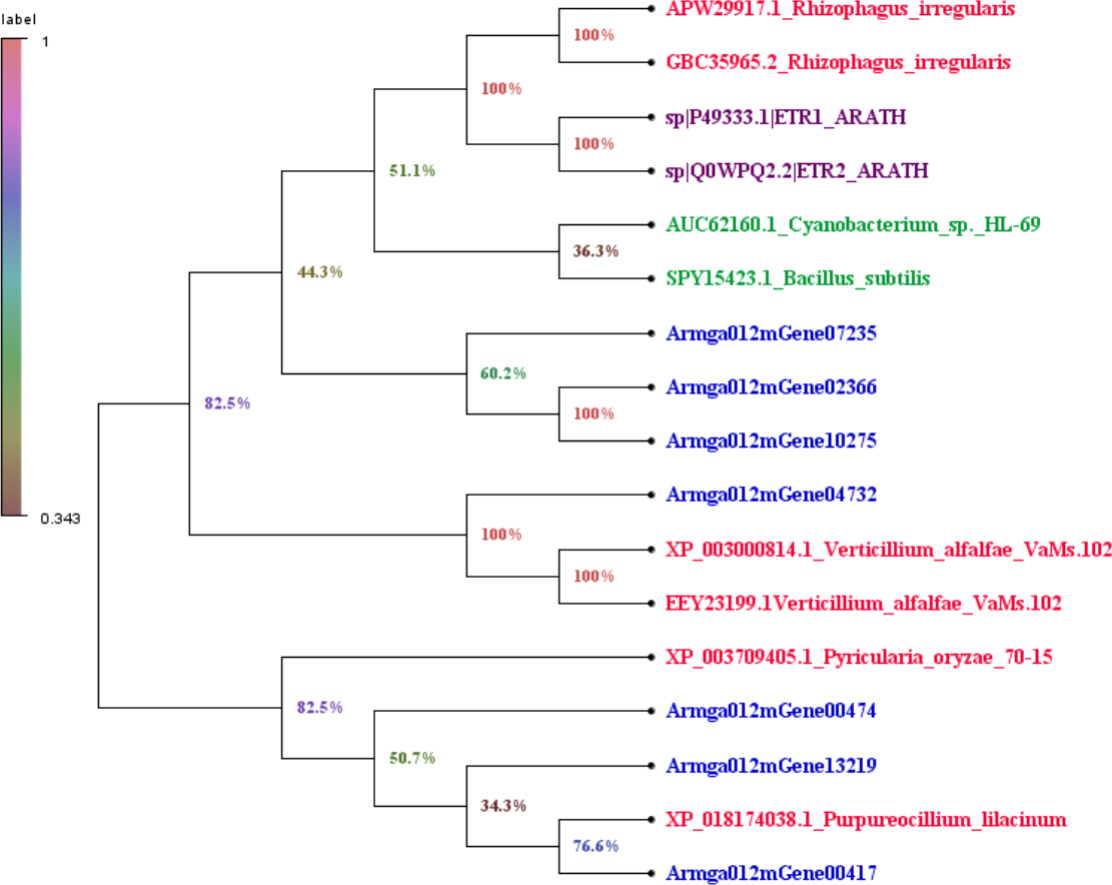
Phylogenetic tree of genes containing ET receptor domain in *A. gallica* 012m with other seven species. The topology of the phylogenetic tree was constructed by the maximum likelihood method.

We downloaded the sequence information of ET receptors in fungi, bacteria, *Arabidopsis thaliana* from GenBank with a total of 10 sequences (File S3). Finally, by comparing 7 species with the ET receptor domain of *A. gallica* 012m, an analysis of the phylogenetic relationship is shown in Fig. 7. The result showed that *A. gallica* 012m (Armgao012mGene04732) had a high homology correlation with *Verticillium alfalfae VaMs.102* (GenBank: XP_003000814.1, EEY23199.1). What’s more, *A. gallica* 012m (Armgao012mGene00417) had homology with *Purpureocillum lilacinum* (GenBank: XP_018174038.1). The phylogenetic tree analysis inferred that *A. gallica* 012m might possess ET receptor domains.

## Discussion

ET is generally considered as the senescence plant hormone, and inhibits the growth process of plants (Chague et al., 2006; Pierik et al., 2006). However, ET has inhibitory and stimulatory effects on plant growth depending on the concentration (Iqbal et al., 2017). ET is reported to inhibit root elongation through interaction with auxins (Muday, Rahman & Binder, 2012), while root elongation of some plants including rice, rye, tomato and white mustard were stimulated by low levels of ET. Abts *etc.* reported that ET regulated early root growth in a dose-dependent manner (Abts et al., 2014). Khan *etc.* reported that ETH could increase the leaf area of mustard at a lower concentration, while inhibiting at higher concentration (Khan et al., 2008). In the liquid culture of *A. gallica* 012m, ETH-5 ppm decreased the biomass of mycelium extremely significantly, while ETH−0.1 ppm enhanced the biomass of mycelium extremely significantly and ETH-2 ppm enhanced the biomass of mycelium significantly. On the solid plate, ETH−0.1 ppm and ETH-2 ppm improved the mycelial growth, while ETH-5 ppm inhibit it. Altogether *A. gallica* 012m showed similar dose-dependent responses to ET like the way of plants above. Different transcriptional profiles of mycelia cultured in exiguous ETH and non-ETH media were carried out. The results showed that the number of up-regulated or down-regulated genes are increased along with the concentrations of ETH. Half of up-regulated or down-regulated genes of ETH−0.1 ppm and ETH-2 ppm coincided with ETH-5 ppm. However, it is hard to explain the great difference of DEGs between ETH−0.1 ppm and ETH-2 ppm.

Several DEGs related to the growth of A. gallica 012m were predicted by by using SMART platform. The up-regulated Pyr_redox gene had been found in bacteria, fungi and yeast as TRX (thioredoxin) system, which is one of the main antioxidant systems responsible for maintaining cellular redox homeostasis and essential for cellular viability (Missall & Lodge, 2005; Oliveira et al., 2010; Marshall et al., 2019). Budding yeast contains TRR1, which encodes the cytoplasmic thioredoxin reductase that reduces the oxidized disulfide form of TRX for the protection of yeast cells against oxidative and reductive stress (Singh, Kang & Park, 2008). The up-regulated HMG domains are known as members of the HMG superfamily and typically bind to DNA (Štros, Launholt & Grasser, 2007). Some HMG box proteins have been identified in fungi, including *Saccharomyces cerevisiae* (Ray & Grove, 2012), *Aspergillus nidulans* (Karácsony et al., 2014), *Schizosaccharomyces pombe* (Albert et al., 2013) and *Podospora anserine* (Dequard-Chablat & Alland, 2002), which have various functions. Yoshihara *etc.* had proposed that the new N. crassa KO strain mhg1KO, which is a protein with HMG domain, showing a short-lifespan. Therefore, its hyphal growth ceased after about two weeks of cultivation, while the wild-type continuing for over two years (Yoshihara et al., 2017). The results implied up-regulated HMG gene might improve the growth of mycelia. GAL4p (C6 zinc finger proteins) belong to the zinc cluster family, which is the largest fungal-specific TF (transcription factors) family (Ekaterina, 2017; Cho & Park, 2022). AflR, as a Gal4p, plays essential roles in fungal development and regulates secondary metabolism in *A. flavus*. RosA, a GAL4-like Zn2Cys6 transcription factor, inhibits sexual development in *A. nidulans* (Vienken, Scherer & Fischer, 2005). In the ETH−0.1 ppm and ETH-2ppm, the growth of mycelia might benefit from the up-regulated expression of Pyr_redox, GAL4P and HMG genes.

ZnF_C2H2 (C2H2 zinc finger) proteins, as a major class of transcription factors, have been functionally validated in fungal growth, development, stress responses, metabolism, sexual reproduction and virulence (Xiong et al., 2015). In *Metarhizium acridum*, MaNCP1 (metarhizium acridum nitrate-related conidiation pattern shift regulatory factor 1), as a C2H2 zinc finger protein, was involved in governing nitrogen utilization and conidial yield (Li, Xia & Jin, 2022). Ribosomal protein S4 is a protein of the small ribosomal subunit involved in protein synthesis (Lu et al., 2015). In *S. cerevisiae*, mutant of S4 ribosomal proteins lead to telomere length (Askree et al., 2004), hydrogen peroxide sensitivity and modified neomycin sulfate sensitivity (Parsons et al., 2006). In the research of *Candida albicans*, cDNA microarray analysis of null mutants showed that carbohydrate and nitrogen metabolic processes were repressed. In the ETH-5 ppm, the growth of mycelia might be impaired by the down-regulated expression of ZnF_C2H2 protein which affects the transcription process and ribosomal protein S4 protein which inhibits the carbohydrate and nitrogen metabolic processes.

Those DEGs are more likely to explain the significant effect with different concentrations of ETH on the growth of *A. gallica* 012m to some extent. We still feel regrettable that the mechanism of A. gallica 012m response to ET could not be fully elucidated because many top 10 DEGs cannot be annotated properly. What is the role ET in the interaction between *G. elata* and *A. gallica* 012m? It was presumed that *G. elata* need some kinds of signaling molecules to guide the *A. gallica* growth towards itself for energy and nutrition. We speculated that ET is a kind of signaling molecule, which help *A. gallica* capture mycelia. On the other hand, ET is the signaling molecule, which guides *A. gallica* to live plant.

The RNA-seq analysis indicated that ETH treatment influenced the gene expression of *A. gallica* 012 m significantly, and implied that ET could be the signaling molecule of *A. gallica* 012m. As signaling molecule, ET receptors and its signaling pathway in plants had been well studied (Merchante, Alonso & Stepanova, 2013; Shakeel et al., 2013; Bakshi et al., 2015). In bacteria, most of the researches concerning ethylene receptors were obtained from studies on the *Cyanobacterium synechocytis* (Bidon et al., 2020; Carlew, Allen & Binder, 2020). Lacey & Binder (2016) first demonstrated that *Synechocystis* Ethylene Response1 (SynEtr1) from *Synechocystis sp*. *PCC6803*, as a biofunctional receptor responses to light and ethylene, contains ET binding domains. Recent genomic data revealed many putative ET receptors in nonplant species including bacteria, fungi and animals (Carlew, Allen & Binder, 2020). There were three transmembrane helices with seven conserved amino-acids including GAF, HK, HA, PAS/PAC, R, P, and STYKc (Papon & Binder, 2019). ET receptors homologs were also predicted in genomes of early diverging fungi which used to be symbiont with plant or colonize decaying plant (Herivaux et al., 2017). Seven ET receptors of *A. gallica* 012m were predicted based on RNA-seq and genome. There were 7 conserved amino-acids including GAF, Hiska, HATPase_c, PAS, HAMP, REC and STYKc. However, only Armga012mGene13219 possessed five transmembrane helices. We speculated that there were ET receptors in *A. gallica* 012m.

It had been demonstrated that ET involved in the plant-fungi interaction. Therefore, some early diverging fungi, known to behave as plant symbionts, were found in ET receptors homologs (Papon & Binder, 2019). Our work provided a new perspective of the hormonal communication that might operate in these symbioses, and ET might play an important role in the process.

## Conclusions

In conclusion, a low concentration of exogenous ETH improved the growth of mycelia, while a high concentration of exogenous ETH inhibited the growth of mycelia in both solid and liquid media. The RNA-seq analyses showed that the number of up-regulated or down-regulated genes are increased along with the concentrations of ETH. Based on the structure prediction of DEGs, the growth of mycelia might benefit from the up-regulated expression of Pyr_redox, GAL4 and HMG genes in the ETH−0.1 ppm and ETH-2 ppm. Therefore, the growth of mycelia might be impaired by the down-regulated expression of ZnF_C2H2 and ribosomal protein S4 proteins. Those DEGs are more likely to explain the significant effect with different concentrations of ETH on the growth of *A. gallica* 012m to some extent.We speculated that *A. gallica* 012m contains seven ET receptor protein domains. Based on cluster analysis and comparative studies of proteins, the result showed that putative ET receptor domains of *A. gallica* 012m have a higher homologous correlation with fungi. Eventually, we speculate that ET receptors exist in *A. gallica* 012m.

##  Supplemental Information

10.7717/peerj.14714/supp-1 Supplemental Information 1 *T*-test for Equality of Means

10.7717/peerj.14714/supp-2 Supplemental Information 2 The DEGs of ETH−0.1 ppm were analysed by the R package “edgeR”

10.7717/peerj.14714/supp-3 Supplemental Information 3 The DEGs of ETH-2 ppm were analysed by the R package “edgeR”

10.7717/peerj.14714/supp-4 Supplemental Information 4 The DEGs of ETH-5 ppm were analysed by the R package “edgeR”

10.7717/peerj.14714/supp-5 Supplemental Information 5 Annotation results of top10 of upregulated genes and downregulated genes under ETH−0.1 ppm

10.7717/peerj.14714/supp-6 Supplemental Information 6 Annotation results of top10 of upregulated genes and downregulated genes under ETH−2.0 ppm

10.7717/peerj.14714/supp-7 Supplemental Information 7 Annotation results of top10 of upregulated genes and downregulated genes under ETH−5.0 ppm

10.7717/peerj.14714/supp-8 Supplemental Information 8 ET receptor protein sequence of fungi from NCBI

10.7717/peerj.14714/supp-9 Supplemental Information 9 Screened candidate ethylene receptors

10.7717/peerj.14714/supp-10 Supplemental Information 10 Genes containing ET receptor domain in *A. gallica* 012m with other 7 species

10.7717/peerj.14714/supp-11 Supplemental Information 11 Pictures for Table 2

## Additional Information and Declarations

### Data Availability

The following information was supplied regarding data availability: Data is available at the SRA: PRJNA759758.
