# Supplementary material for: Comparative transcriptome analysis of Armillaria gallica 012m in response to ethephon treatment
Source: PeerJ. 2023 Jan 17;11:e14714. doi: 10.7717/peerj.14714 (PMC10088873; doi:10.7717/peerj.14714)
Supplement: Supplemental Information 1 [file peerj-11-14714-s001.docx]

| **Table S1 T-test for Equality of Means** | | | | | | |  |
| --- | --- | --- | --- | --- | --- | --- | --- |
| Group | Mean Difference | Std. Error Difference | 95% Confidence Interval of the Difference | | Sig  (2-tailed) | Level | Biomass increased percentage |
|  |  |  | Lower | Upper |  |  |  |
| ETH-0.1ppm vs. BK | 0.4349 | 0.0605 | 0.2669 | 0.6029 | 0.002 | Extreme significant | 88.0±9.1% |
| ETH-2ppm vs. BK | 0.3408 | 0.0288 | 0.2610 | 0.4289 | <0.001 | Extreme significant | 66.1±7.9% |
| ETH-5ppm vs. BK | -0.4287 | 0.0187 | 0.4805 | 0.3769 | <0.001 | Extreme significant | -86.8±5% |
