# Supplementary figures and images for: Comparative transcriptome analysis of Armillaria gallica 012m in response to ethephon treatment
Source: PeerJ. 2023 Jan 17;11:e14714. doi: 10.7717/peerj.14714 (PMC10088873; doi:10.7717/peerj.14714)

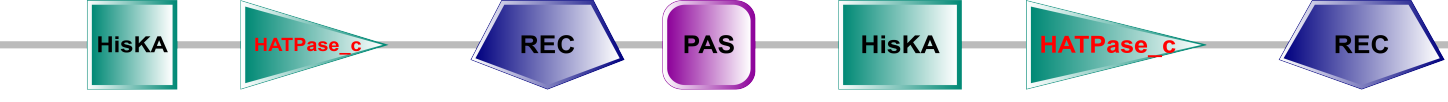

Supplement: Supplemental Information 11 [file peerj-11-14714-s011.zip › Armga012mGene00417.png]

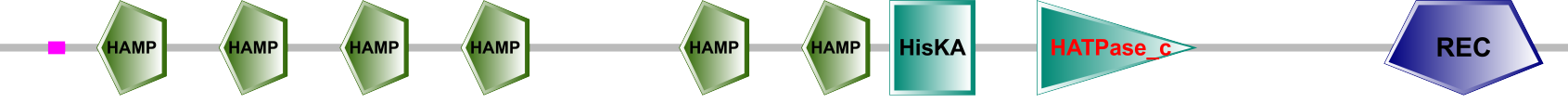

Supplement: Supplemental Information 11 [file peerj-11-14714-s011.zip › Armga012mGene02366.png]

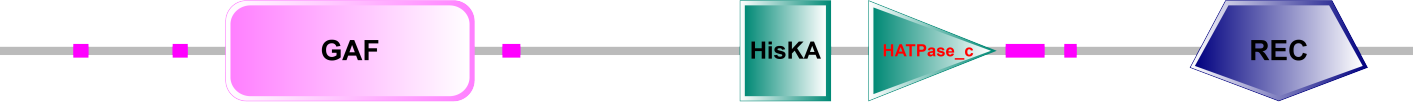

Supplement: Supplemental Information 11 [file peerj-11-14714-s011.zip › Armga012mGene04732.png]

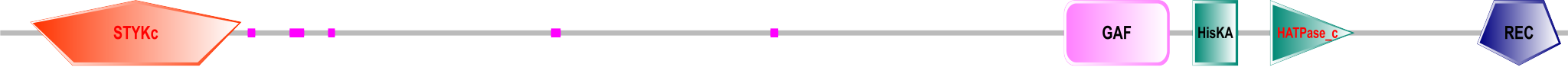

Supplement: Supplemental Information 11 [file peerj-11-14714-s011.zip › Armga012mGene07235.png]

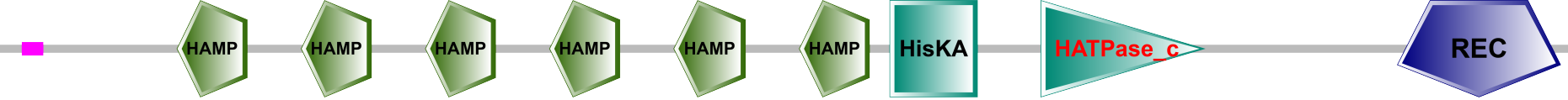

Supplement: Supplemental Information 11 [file peerj-11-14714-s011.zip › Armga012mGene10275.png]

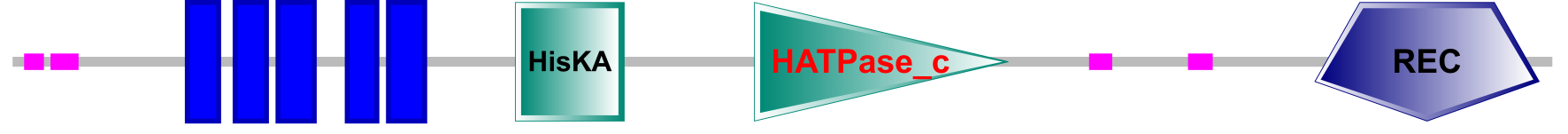

Supplement: Supplemental Information 11 [file peerj-11-14714-s011.zip › Armga012mGene13219.png]

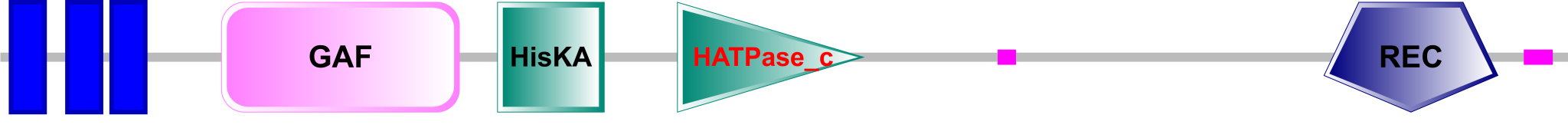

Supplement: Supplemental Information 11 [file peerj-11-14714-s011.zip › SMART_protein_schematic.png]

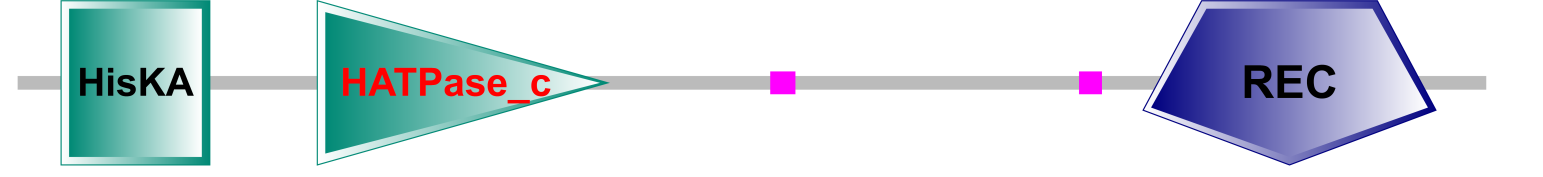

Supplement: Supplemental Information 11 [file peerj-11-14714-s011.zip › Verticillium alfalfae.png]

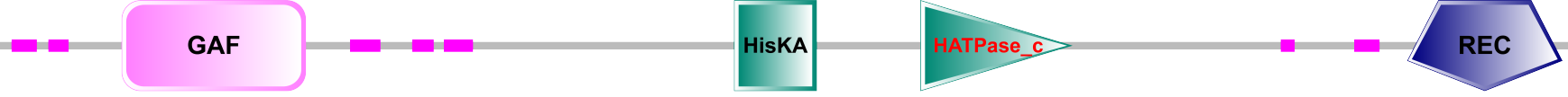

Supplement: Supplemental Information 11 [file peerj-11-14714-s011.zip › _Armga012mGene00474.png]

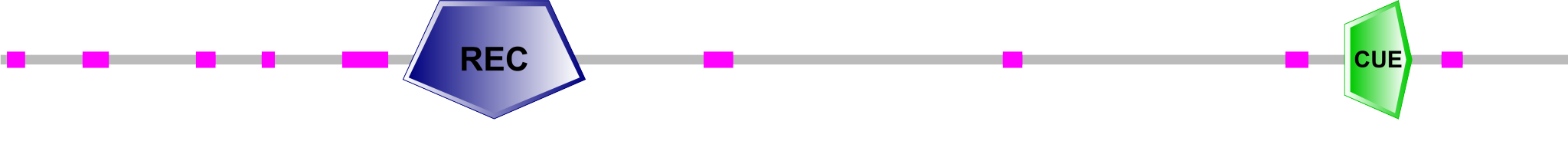

Supplement: Supplemental Information 11 [file peerj-11-14714-s011.zip › _Armga012mGene24744.png]

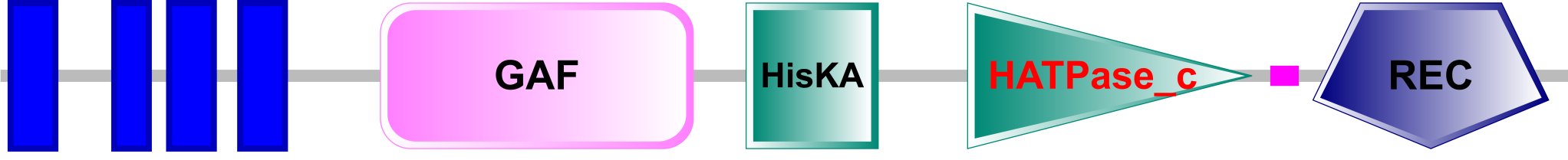

Supplement: Supplemental Information 11 [file peerj-11-14714-s011.zip › arabidopsis thaliana.png]
